# Pathway-specific modulatory effects of neuromuscular electrical stimulation during pedaling in chronic stroke survivors

**DOI:** 10.1186/s12984-019-0614-9

**Published:** 2019-11-19

**Authors:** Shi-Chun Bao, Wing-Cheong Leung, Vincent C. K. Cheung, Ping Zhou, Kai-Yu Tong

**Affiliations:** 1 Department of Biomedical Engineering, The Chinese University of Hong Kong, Hong Kong, China; 2 Brain and Mind Institute, The Chinese University of Hong Kong, Hong Kong, China; 3 School of Biomedical Sciences, and The Gerald Choa Neuroscience Centre, The Chinese University of Hong Kong, Hong Kong, China; 4 The KIZ-CUHK Joint Laboratory of Bioresources and Molecular Research of Common Diseases, The Chinese University of Hong Kong, Hong Kong, China; 5 Department of Physical Medicine and Rehabilitation, The University of Texas Health Science Center at Houston, Houston, 77030 TX USA; 6 TIRR Memorial Hermann Research Center, Houston, 77030 TX USA

**Keywords:** NMES, Pedaling, Cortico-muscular coupling, Generalized partial directed coherence, Stroke, EEG/EMG

## Abstract

**Background:**

Neuromuscular electrical stimulation (NMES) is extensively used in stroke motor rehabilitation. How it promotes motor recovery remains only partially understood. NMES could change muscular properties, produce altered sensory inputs, and modulate fluctuations of cortical activities; but the potential contribution from cortico-muscular couplings during NMES synchronized with dynamic movement has rarely been discussed.

**Method:**

We investigated cortico-muscular interactions during passive, active, and NMES rhythmic pedaling in healthy subjects and chronic stroke survivors. EEG (128 channels), EMG (4 unilateral lower limb muscles) and movement parameters were measured during 3 sessions of constant-speed pedaling. Sensory-level NMES (20 mA) was applied to the muscles, and cyclic stimulation patterns were synchronized with the EMG during pedaling cycles. Adaptive mixture independent component analysis was utilized to determine the movement-related electro-cortical sources and the source dipole clusters. A directed cortico-muscular coupling analysis was conducted between representative source clusters and the EMGs using generalized partial directed coherence (GPDC). The bidirectional GPDC was compared across muscles and pedaling sessions for post-stroke and healthy subjects.

**Results:**

Directed cortico-muscular coupling of NMES cycling was more similar to that of active pedaling than to that of passive pedaling for the tested muscles. For healthy subjects, sensory-level NMES could modulate GPDC of both ascending and descending pathways. Whereas for stroke survivors, NMES could modulate GPDC of only the ascending pathways.

**Conclusions:**

By clarifying how NMES influences neuromuscular control during pedaling in healthy and post-stroke subjects, our results indicate the potential limitation of sensory-level NMES in promoting sensorimotor recovery in chronic stroke survivors.

## Background

Stroke is a cardiovascular disease with high mortality and disability. It is one of the leading causes of death worldwide. More than 11 million stroke cases were reported every year in China, and more stroke incidences are expected due to the coming aging population [1]. 80% of stroke survivors suffered from motor impairment contralateral to the lesioned hemisphere. Their functional mobility and independence were reduced after stroke, which severely decreased their life quality. Hemiparesis is one of the typical symptoms which would decrease gait performance after stroke [2]. Despite the spontaneous recovery within the first few weeks after stroke onset, effective motor rehabilitation is essential to facilitate motor relearning and neuroplasticity, especially for chronic stroke patients with more than six-month brain injury [3].

Neuromuscular electrical stimulation (NMES) utilizes short electrical pulses to activate the peripheral nerves and to induce peripheral limb movement by modulating neuron hyperpolarization or depolarization [4]. For stroke survivors, their spinal motor neurons are intact and excitable despite cortical impairments. NMES has been extensively applied in motor recovery of various neurological disorders, such as stroke [5], spinal cord injury [6], cerebral palsy [7], and so forth. Lower limb locomotion is indispensable for the daily life of human beings. NMES has also been applied in lower limb rhythmic gait training for hemiplegic patients [8]. Nevertheless, it is challenging for stroke patients with severe motor impairments to support themselves, maintain balance, and walk coordinately. Stationary pedaling might be a suitable alternative as pedaling and walking share similar activation patterns of muscle groups [9]. Synchronized NMES and pedaling have been investigated for stroke survivors. It was demonstrated that NMES pedaling enhanced lower limb motor functions for stroke patients, and improved muscle activation and symmetrical involvement [10, 11].

Despite the muscular changes, NMES might also activate afferent sensory input to the central motoneurons and facilitate motor relearning of cortico-spinal excitability by modulating the synaptic neuron properties [12]. NMES results in plastic changes within cortical motor structures [13] and further promotes neuroplasticity for stroke survivors [14, 15]. fMRI study also confirmed the functional changes of the sensorimotor cortex in voluntary plantar flexion tasks with NMES [16]. The cortical event-related desynchronization patterns during lower limb movement with NMES were similar to that of active movement instead of passive movement [17]. The beta band cortico-muscular coherence was modulated by peripheral stimulation [18, 19]. Moreover, after 8-weeks combined NMES and motor training for stroke patients, the cortico-muscular coherence in the NMES group was significantly higher when compared with the control group [20]. However, these studies investigated pre- and post-NMES difference considering mainly static or semi-static locomotion activities such as isometric contraction, so the functional role of NMES in complex dynamic activities like pedaling was still rarely explored. NMES in dynamic movement environment might help understand neuromuscular control mechanisms during simultaneous electrical stimulation in the physical world and might contribute to NMES-based clinical applications. Conventional channel level EEG analysis cannot identify locally coherent cortical field activities from the scalp EEG signals, which are easily influenced by artifacts, volume conduction, and other non-brain signals [21]. Blind-source separation strategies like independent component analysis could effectively remove non-physiological artifacts and decrease the volume conduction effects [22, 23].

Previous studies have shed light on the specific cortical regions involved in different rhythmic movement tasks with electroencephalogram (EEG) [24, 25], the time-frequency cortical response patterns of active or passive movement [24], and also the interaction between EEG and electromyography (EMG) signals [26]. As far as we know, few studies explored the NMES modulatory effects in dynamic movement. It is shown that NMES pedaling induced reactivation of sensorimotor functions for incomplete spinal cord injury patients [27]. However, the analysis from the aspect of cortical and muscular interaction remains limited, understanding the neuromuscular control process might promote poststroke rehabilitation [28]. Cortico-muscular coupling has been probed in acute, subacute, and chronic stroke patients [29], and might serve as a neurophysiological measure of functional relevant contribution of reorganized cortical area to motor recovery [30]. It is pointed out that human ascending afferent feedback pathways also contribute to cortico-muscular coupling, not just descending pathways [31]. Directed cortico-muscular coupling illustrated causality relationship within the sensorimotor loops, can reflect not only the motor commands of descending pathways, but also the ascending sensory pathways from muscle to the brain [32, 33]. Such descending and ascending pathway indicate respectively motor and sensory feedback integration within the motor control process [34]. The parallel recovery of both sensory and motor system is critical for functional recovery after stroke [35]. Manipulation of peripheral neural pathways could modulate human cortico-muscular coupling [36] and reflex regulation during phasic leg cycling activities for spinal cord injury patients [37]. Additionally, NMES induced increased directed causality from the primary motor cortex to the primary somatosensory cortex [38].

Nevertheless, it remains an open question for how NMES modulates the closed-loop sensorimotor control process for healthy subjects and stroke patients. Understanding the functional role of NMES in directional cortico-muscular coupling might help explain the pathway-specific modulatory effects of NMES in neuroplasticity and the closed-loop motor control process [39]. For healthy subjects, NMES above the motor threshold tended to increase the excitability of the corticomotor pathway [40]. Moreover, the voluntary movement involvement with NMES can promote larger cortical excitability than the stimulation alone, which was evidenced by motor evoked potentials (MEPs) [41].

In this study, the short-term, within-session cortico-muscular contribution during active, passive, and sensory-level NMES pedaling sessions are analyzed and compared for both healthy and chronic stroke subjects with moderate lower limb functions. We hypothesized that NMES on the paretic lower limb could modulate directed cortico-muscular coupling during rhythmic pedaling, and the cortico-muscular coupling during NMES pedaling might be similar to that of active pedaling. But the modulatory effects could be different between healthy subjects and chronic stroke survivors.

## Methods

### Subjects

Sixteen chronic stroke survivors with moderate lower extremity motor functions were randomly a large pool of stroke subjects considering the subjects’ and experimental schedules. Clinical assessments were conducted by well-trained assessors who were blinded to the whole experiment settings. Demographic information is shown in Table 1. All stroke subjects experienced a first-ever unilateral stroke (9 females; age: 58.5 ± 10.3 years; time since stroke: 5.5 ± 3.6 years; FMA-LM motor function score: 22.0 ± 4.4, range: 14-28, full mark: 34; affected leg side: 8 right; ischemic: 10), lesion site information was retrieved from clinical records. Additionally, twelve healthy subjects (all male, age: 25.4 ± 5.1 years) participated in the study. No history of neuromuscular disorders or lower limb injury within the recent two years was reported for both the healthy and stroke survivors.

**Table 1:** Demographic information of the subjects

Stroke								Healthy		
Subject	Age	Gender	TSS	Type	FMA-LE	Affected	Lesion Site	Subject	Age	Gender
1	59	F	7.3	H	24	R	Cortical L	17	22	M
2	59	F	6.3	H	20	R	Subcortical L	18	20	M
3	60	F	7.4	I	25	R	Subcortical L	19	29	M
4	54	F	14.8	I	26	L	Cortical R	20	26	M
5	69	M	10.4	I	26	L	Cortical R	21	39	M
6	37	M	4.6	I	21	R	Cortical L	22	23	M
7	72	F	5.8	I	25	R	Subcortical L	23	22	M
8	63	M	2.2	I	17	L	Cortical R	24	22	M
9	57	M	2.3	I	21	L	Subcortical R	25	29	M
10	72	F	4.2	I	25	L	Cortical R	26	25	M
11	61	M	9.0	I	28	L	Subcortical R	27	23	M
12	61	M	3.4	H	28	R	Subcortical L	28	25	M
13	65	F	3.9	H	16	L	Cortical R			
14	60	F	2.9	I	20	R	Subcortical L			
15	36	F	2.1	H	17	R	Cortical L			
16	51	M	2.1	H	14	L	Subcortical R			

F=Female; M=Male; H= Hemorrhage; I= Ischemic; R= Right; L= Left; FMA-LE, Fugl-Meyer Assessment Lower Extremity, only motor part, full: 34; TSS, Time since stroke (years). Subject 1-16, chronic stroke, subject 17-28, healthy

### Experimental paradigms

**Fig. 1:**
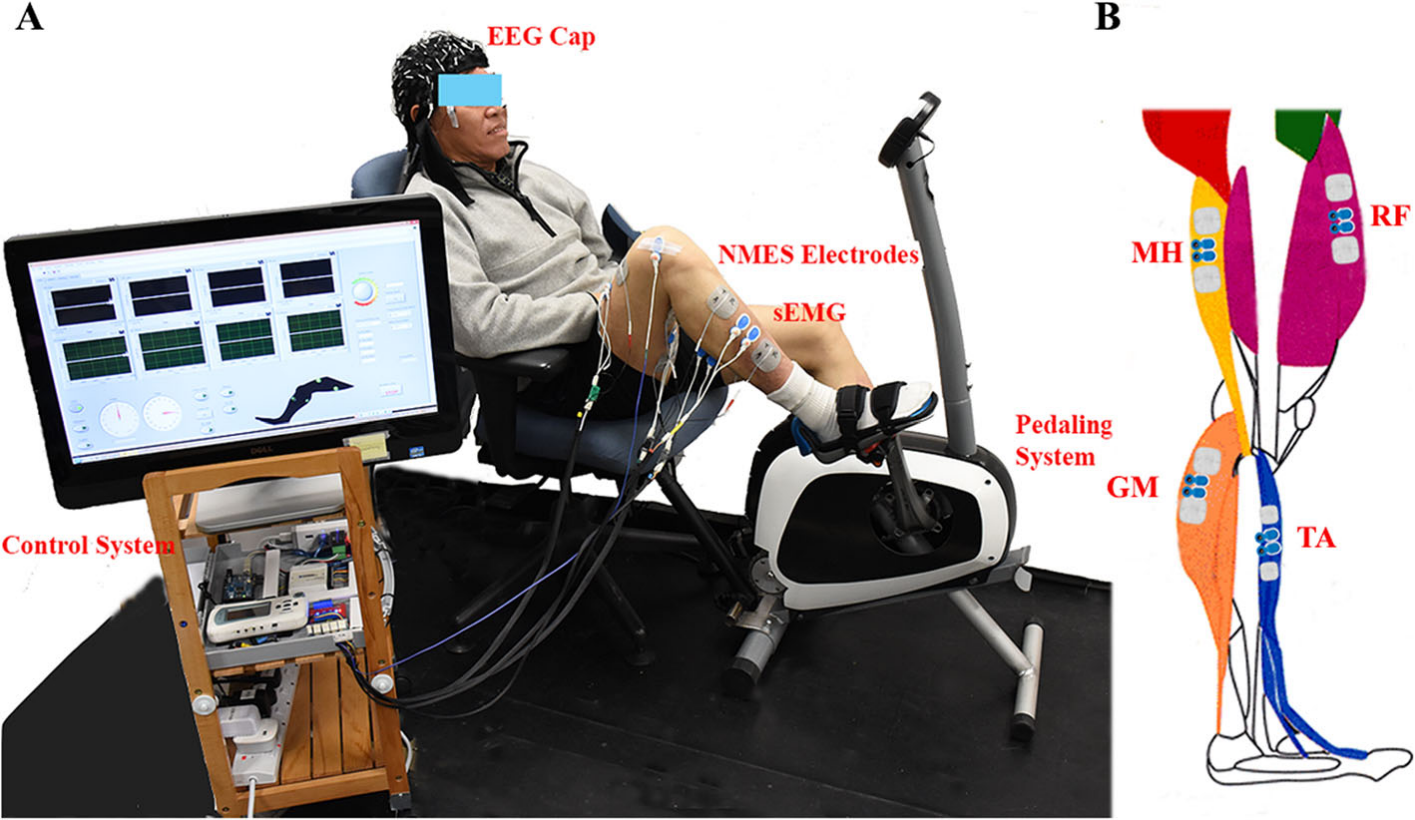
EEG and NMES-pedaling system. **a**, The general structure of the systems involved, including stationary NMES-pedaling system, control system and EEG/sEMG measurement system. **b**, The placement of NMES electrodes and surface EMG electrodes.

The principal measurement system included a Neuroscan EEG measurement system (SynAmps2, Neuroscan Inc, Herndon, USA), and a self-modified stationary bike with adjustable armchair. The detailed information was reported previously [42], as demonstrated in Fig. 1a. Step motor (UIM241 controller, UIRobot Inc, Shanghai, China) with a closed-loop motor controller was mounted near the crank to acquire stable pedaling angle, and a planetary gear was employed to provide power for the pedaling system. Torque sensors (HLT132, Hualiteng Inc, Shenzhen, China) were mounted on the cranks to measure torque balance between the two cranks.

Surface EMG signals of the affected lower limb were obtained with a NI PCIe-6320 DAQ (National Instruments, Austin, USA) and a self-developed amplifier (gain: 1000, input impedance: 10 G *Ω*, 1 kHz sample rate, bandwidth: 0-380 Hz) [43]. Typical muscles included Medial hamstring (MH, knee flexion), Rectus femoris (RF, knee extension), Gastrocnemius (GM, ankle plantarflexion), and Tibialis anterior (TA, ankle dorsiflexion), see Fig. 1b. The EMG electrodes (BlueSensor N, Ambu, Denmark) were placed on the skin surface of the muscle belly of selected muscles with around 2 cm inter-electrode distance. The EMG signal quality was carefully checked before experiments, and good contact was maintained during pedaling. A portable four-channel programmable NMES device (Easy Walker P2-9632, FineCure, Guangzhou, China) was used to stimulate the paretic lower limb muscles with bipolar 50x50 mm NMES surface electrodes (PALS Platinum, Axelgaard Co., CA, USA) near the EMG channels as mentioned before. NMES was only applied in the NMES pedaling session with 40 Hz stimulation frequency for 10 min during 25 RPM pedaling. The pulse width was 420 *μ*s, and the amplitude was 20 mA. Such intensity is below the motor threshold to excite the muscles [44] and is sensory-level stimulation in our pre-experiment testing. The NMES device was controlled by an Arduino Board (Arduino Mega 2560 V3.1, Arduino Corp., Italy). A four-channel solid-state relay module (GTGM04) was used to reduce the data transmission delay. The cyclic stimulation pattern was defined according to the averaged EMG profiles during constant speed pedaling in a classical study [45], for example, TA stimulation was applied in the cycle phase of 260-340 ^∘^.

One computer was used for EEG recording with Curry 7 software (Neuroscan Inc, Herndon, USA). Another computer with Labview 2015 (National Instruments, Austin, USA) based user interface was employed to control the whole pedaling system, measure the EMG signals, and collect the pedaling torque and crank angle with 1 kHz sample rate. Parallel port was used to synchronize the EEG computer and pedaling control computer by sending trigger commands at the start and the end of the pedaling sessions with Labview embedded m-scripts, the time difference between the two computers was maintained within 1 ms.

All experiments were conducted in a radiofrequency-shield room (Universal Shielding Corp., NY, USA). Subjects were required to sit comfortably on the armchair and to lean their head against the backrest of the chair, with their feet fixed to the pedaling cranks. Large head motion was not allowed during experimental sessions. No visual access to the pedaling process was allowed to prevent possible movement observation and vision impacts. The surrounding environment was kept constant to avoid confounding effects. 128-channel Quick-Cap (Neuroscan Inc, Herndon, USA) was used to measure EEG signals during pedaling sessions with 500 Hz sample rate, the reference electrode was placed close to the middle central scalp, and impedance was kept under 5 k *Ω*.

Each subject performed three sessions of pedaling experiments on the same day with a 10-min intermediate break [17]. To prevent the carry-over effects and muscle fatigue of active and NMES pedaling [46], the pedaling order was passive, active, and NMES pedaling in sequence. Subjects were asked to pedal with volitional forces during active pedaling using their affected limbs for stroke survivors and left legs for healthy controls, and to relax and follow the crank movement during passive pedaling and NMES pedaling, while NMES was only applied during NMES pedaling session. Pre-experiment tuning and warm-up were performed to guarantee stable pedaling and effective muscle response in the whole pedal cycle. The maximum knee extension angle was maintained about 140 ^∘^-150 ^∘^ for both legs by adjusting the distance between the armchair and pedaling system using a goniometer. Subjects started with left leg or affected leg at the position with a crank angle of 0/360 ^∘^ (top dead center, TDC) for each session. Each experimental session lasted around 12 min. A Labview control system was used to control the pedaling speed, and the first and last minutes were the low-speed periods with 15 revolutions per minute (RPM). Only the middle 10-min constant high speed pedaling (25 RPM) period was utilized for further analysis.

### Signal processing

**Fig. 2:**
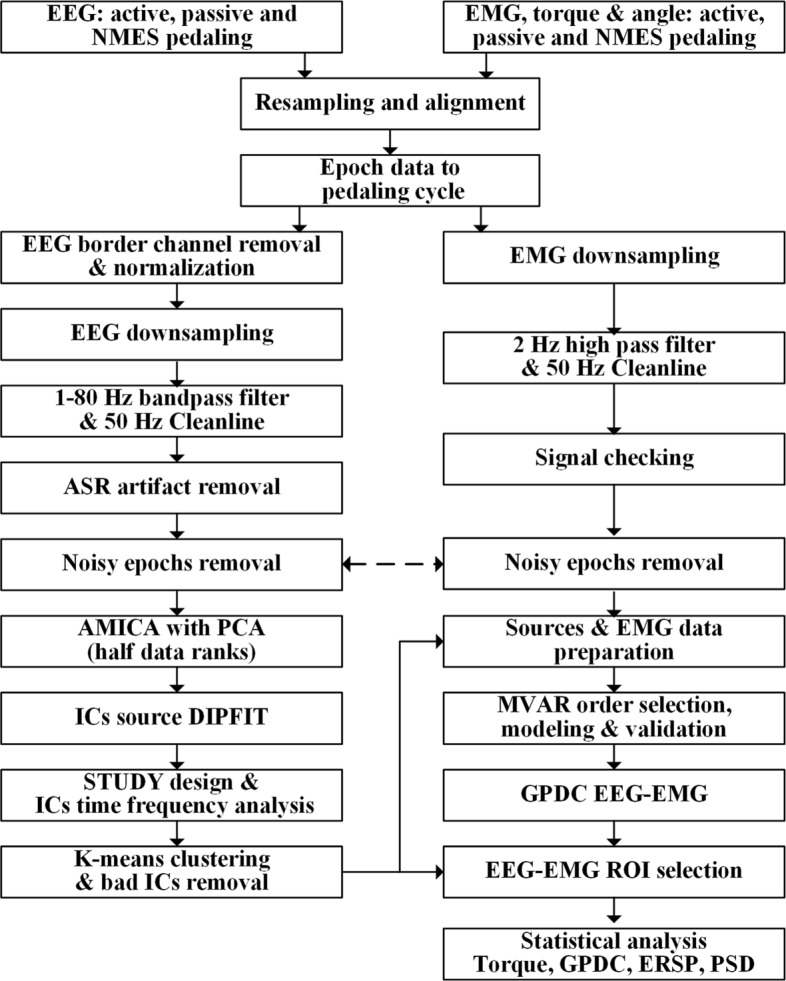
General signal processing flow charts. All the pre-processing, cleaning up and analysis techniques were included

EEG/EMG signal processing was conducted with self-written Matlab scripts based on the signal processing, EEGLAB, and related Matlab toolbox [22]. The general procedures of the EEG/EMG analysis are presented in Fig. 2, the procedures followed the general EEGLAB source localization procedures and fulfilled the latest technique updates [26, 47]. First of all, the timelines of EEG, EMG, torque, and angle signals were aligned, only the 25 RPM pedaling periods were selected. EEG events were carefully updated following pedaling phase angles, and the epoch length was equal to the pedal cycle duration (2.4 s), starting from 0/360 ^∘^ TDC position. As for the 128 channel EEG-cap, channels near the peripheral border and bilateral ears (such as T9, FT9, and so forth) were deselected due to unstable EEG gel contact, resulting in 100 EEG channels for further analysis. For stroke subjects, to make the lesion site consistent, the ipsilesional hemisphere was standardized to the right brain, the EEG channels were shifted to the opposite side with reference to medial interhemispheric fissure line, and the affected lower limbs were normalized to the contralateral side accordingly [29]. Afterwards, the EEG signal was downsampled to 250 Hz, then bandpass filtered (1-80 Hz) to remove signal drifts and select the frequency up to the gamma band. Furthermore, line noise was removed with CleanLine plugin, then artifact subspace reconstruction (ASR) algorithm was employed based on the cleanest part of the signal which was determined by the algorithm itself to remove noisy channels and correct continuous time series data [48]. EMG signals were downsampled to 250 Hz as well, then filtered with a 2 Hz high pass filter, and line noise was removed with CleanLine plugin. EMG epochs with large fluctuation (around three times of normal EMG fluctuation amplitude) were also removed, as well as the corresponding EEG trials. For each pedaling session, 214.7 ± 20.5 epochs and 239.2 ± 23.1 epochs were acquired for healthy and stroke survivors respectively, with 8.5 ± 4.4 channels and 11.2 ± 6.0 channels being rejected in the ASR process. There were no significant differences between different pedaling protocols for both stroke and healthy subjects. Removed channels were interpolated back to make channel number consistent across sessions and subjects, and EEG signals were further average referenced. With the aforementioned steps, we could acquire relatively clean signals.

Subsequently, a three-model adaptive mixture independent component analysis (AMICA) algorithm was applied to EEG signals to determine the independent components (ICs) during pedaling [49]. Only half of the signal ranks were kept in the AMICA process due to the limited data length, and the independent component analysis could adequately separate sources of activity. Signals whose likelihood in the derived model was more than 3 standard deviations from the median likelihood were removed [23]. Each IC was modeled as an equivalent current dipole with DIPFIT plugin, a boundary element head model based on a standard Montreal Neurological Institute MRI template was used to determine the IC dipole. Only the ICs with best-fit dipole less than 85% of the scalp map variance were considered for further analysis [50]. Additionally, in-brain ICs with less than 15% residual variance were clustered based on dipole location (weight: 10), scalp topography (weight: 2) and power spectrum (weight: 1) across healthy and stroke survivors respectively, the dimensionality of consequent vectors was deduced to 10 with principal component analysis [51]. Vectors were k-means clustered into 14 and 18 clusters for healthy and stroke survivors respectively, and such clusters included the predominant cortical sources during movement, the cluster centroid locations were determined with TalairachClient software [52]. IC clusters with more than half of the subjects were further considered, and outlier components and non-brain ICs were manually removed. The clusters with the largest numbers of ICs and the four-channel surface EMG signals were used for subsequent cortico-muscular coupling analysis for stroke and healthy subjects (Clusters with fewer ICs were hard to perform group comparison).

As for time-frequency analysis of source signals, the event-related spectral perturbation (ERSP) reflects EEG cortical oscillation during pedaling. ERSP was defined as equation 1, where *n* is the number of trials for analysis, and *P*_*k*_(*f*,*t*) is the power spectral estimation of the *k*th trial at frequency *f* and time *t*. Three cycles of wavelet were utilized to perform ERSP analysis in EEGLAB. Additionally, the power spectral density (PSD) was calculated with Pwelch method [22].

1 $$ \text{ERSP}(f, t)=\frac{1}{n} \sum_{k=1}^{n}\left(P_{k}(f, t)^{2}\right)  $$

Regarding the directed cortico-muscular coupling relationship between source level EEG and EMG signals, a generalized partial directed coherence (GPDC) measure was applied. GPDC is a causal measurement tool based on MVAR modeling [53], and it is an extension to conventional partial directed coherence [54] and was reported to be more robust for less clean time series [55]. GPDC was explored previously to measure the directed communication during movement in Parkinson’s disease [56] and cortical myoclonus patients [57]. As the following Eq. (2) shows, **X**(*t*)=(*X*_1_(*t*),*X*_2_(*t*),…,*X*_*M*_(*t*))^*T*^ is the multivariate M-channel process, **ε**(*t*) is white Gaussian noise vector for each channel, **A**(*k*) is an *M*×*M* matrix of model parameters at time step *k*, *p* is the model order of data samples. To speed up the signal processing, only the first 30 source signals from AMICA decomposition and 4 channel EMG signals were included for bidirectional GPDC analysis with EEGLAB SIFT plugin [58]. All the time series signals were detrended and normalized with unit variance before MVAR modeling [47]. The Viera-Morf method with unbiased covariance estimates was applied to determine the MVAR parameters using 1s sliding windows with 0.1 s overlap [53]. The model order *p* is determined by Schwarz-Bayes Criterion (SBC) and Akaike Information Criterion (AIC) [59], and a consistent model order of 40 was obtained by calculating the mean optimal order for all the files. Test of stability and consistency was performed to validate the model reliability. Percent consistency across data was up to around 85% and MVAR model stability scores were almost full for all the session files of healthy and stroke survivors, suggesting that the MVAR modeling could stably resolve predominant cortico-muscular dynamics.

2 $$ \mathbf{X}(t) = \sum_{k =1}^{p} \mathbf{A} (k)\mathbf{ X} (t-k) + \mathbf{\epsilon} (t)  $$

The MVAR coefficient matrix **A**(*k*) could be transformed into frequency domain $\overline {\mathbf {A}} (f)$, where *i* is the imaginary unit and *Δt* is the time resolution, **I** is the corresponding identity matrix, see Eq. 3.

3 $$ \overline{\mathbf{A}} (f) = \mathbf{I} - \sum_{k= 1}^{p} \mathbf{A}_{k} e^{-i 2 \pi \Delta tf}  $$

Afterwards, the directional causality of GPDC from source *n* to channel *m* can be defined with Eq. 4, where $\overline {\mathbf {A}} (f)$ is normalized by the standard deviation of the **ε**(*t*) model residuals.

4 $$ \text{GPDC}_{n \rightarrow m}(f)=\frac{\frac{1}{\sigma_{m}} \overline{\mathbf{A}}_{mn}(f)}{\sqrt{\sum_{k=1}^{M} \frac{1}{\sigma_{k}^{2}} \left| \overline{\mathbf{A}}_{k n}(f)\right|^{2}}}  $$

Eventually, the ICs in the clusters of interest were selected and compared across pedaling conditions for both healthy and stroke survivors. The dynamics of sliding windows were averaged to acquire the mean GPDC properties in a pedaling cycle. The bidirectional max GPDC, ERSP and PSD properties in the frequency range of 8-35 Hz were considered, including typical alpha, beta band and low gamma bands which were typical sensorimotor rhythms related to dynamic movement [25], while higher frequency bands were not included due to substantial NMES artifacts around 40 Hz.

### Statistical analysis

Statistical analyses were conducted with IBM SPSS Statistics 22 software (IBM, Armonk, NY, USA) or EEGLAB built-in statistical functions. Shapiro-Wilk’s test was used to check the data normality distribution. Outliers out of the 95% confidence interval were replaced with the mean values. The significance level was p < 0.05 in the current study.

One-way ANOVA was used to determine the intra-group torque difference between three pedaling protocols. Post-hoc paired t-test was used with Bonferroni correction. 1000 times bootstrapping was conducted to compare the difference between healthy and stroke survivors for given pedaling protocol. Repeated measures ANOVA with two factors, pedaling protocols (active, passive, and NMES), muscles (MH, RF, GM, and TA) was used to assess the GPDC difference across pedaling protocols and muscles for both stroke and healthy subjects (the GPDC comparison between stroke and healthy subjects was not conducted due to the inconsistent dipole locations from the source localization results). The p-value was adjusted with the Greenhouse-Geisser Epsilon method in case of sphericity violation (Mauchly’s test). Paired t-test was utilized for post-hoc analysis with Bonferroni’s correction. For individual muscles, one-way ANOVA was used to compare the intra-group difference. Due to the uneven numbers of IC sources in three protocols, 1000 times bootstrapping was performed to compare the difference. As for cortical ERSP and PSD properties, EEGLAB inherent statistics were applied with 500 times permutation with FDR correction. Additionally, the mean ERSP and PSD in the selected frequency range were compared with SPSS 1000 times bootstrapping.

## Results

### Pedaling kinematics comparison

**Fig. 3:**
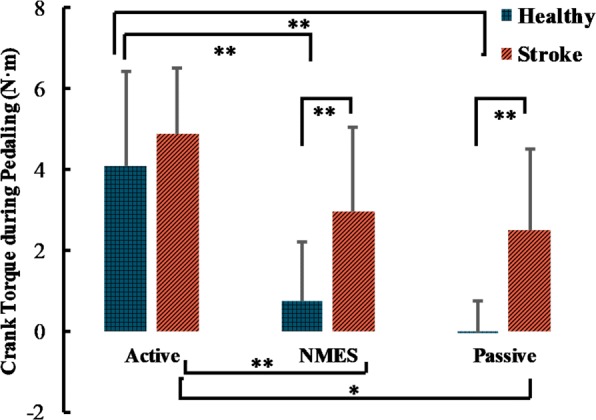
Crank torque values in three pedaling protocols for stroke and healthy subjects, average across subjects, half error-bar= ± 1 SEM., **, *p*<0.01, *, *p*<0.05

One-way ANOVA with Welch correction (variance homogeneity is not satisfied) demonstrated significant main effects of pedaling groups for healthy subjects (*F*_2,18.750_=16.643,*p*<0.001). For stroke survivors, one-way ANOVA found main effects of pedaling protocols as well (*F*_2,45_=6.922,*p*=0.002). Post-hoc t-test reported significant torque difference between active and passive pedaling, active and NMES pedaling for both stroke and healthy subjects, whereas no significant difference was found between NMES and passive pedaling, see Fig. 3. The torque results indicated that the subjects generally conducted the experiments as requested, and the sensory-level NMES did not change the crank balance significantly in pedaling. For specific pedaling protocols, a significant difference of torque values between healthy and stroke survivors was demonstrated for NMES and passive pedaling (*p*=0.007,0.001 respectively), suggesting that the stroke survivors cannot effectively control their lower limb muscles for passive and NMES stimulation tasks.

### Source localization for healthy and stroke subjects

**Fig. 4:**
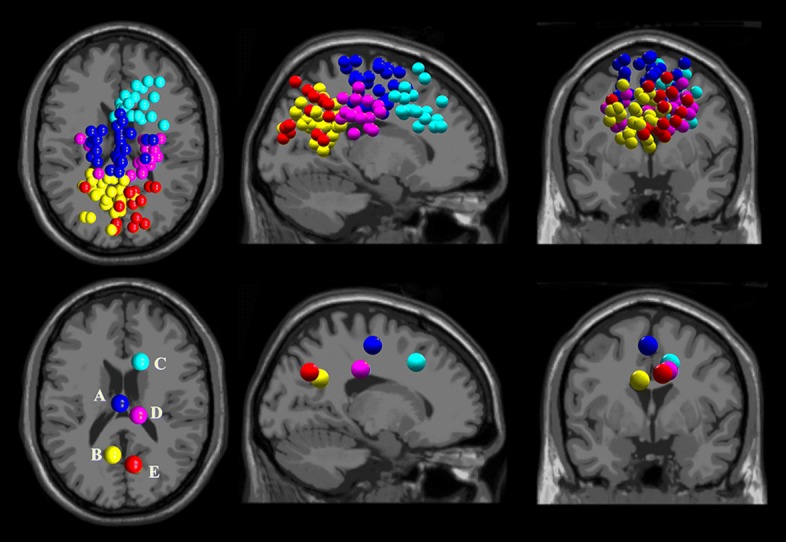
EEG source localization results for twelve healthy subjects during three pedaling protocols. Five independent component clusters were demonstrated with different colors. The top row gives the dipole locations and the bottom row gives the corresponding cluster centroids, the transverse, sagittal and coronal views (from left to right), A-E, different source clusters centroids

**Fig. 5:**
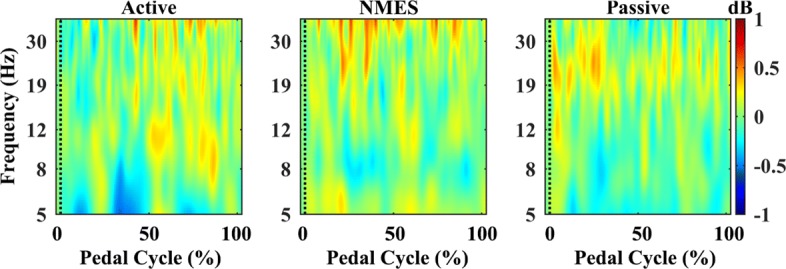
Grand pooled ERSP charts for three pedaling protocols of Cluster A, BA 6 for healthy subjects. Pedal phase dependent cortical responses were observed throughout the pedal cycle (x-axis) and in the selected frequency bands (y-axis, log scaled), warm and cold color indicates increased or decreased cortical activity relative to the mean spectrogram respectively

Only ICs clusters with more than half of the subjects were demonstrated. Across the healthy subjects, five source clusters were identified. The corresponding cluster location, centroids Talairach coordinates [52], Brodmann areas, and numbers of subjects and ICs are illustrated in Table 2. Figure 4 demonstrates all the ICs (upper row) and the identified ICs cluster centroid A-E (lower row), with different color representing different dipole cluster, and the left, middle and right columns respectively being the transverse, sagittal and coronal views. The dipole cluster centroids are located in general close to the middle longitudinal fissure, see Fig. 4, suggesting the functional roles of the middle cortical areas in lower limb pedaling. Cluster A (BA 6) is located in the medial frontal gyrus, near the premotor cortex and supplementary motor area. Cluster A included the largest numbers of subjects and ICs. The grand pooled ERSP plots of BA 6 showed pedal cycle-dependent cortical oscillations shift throughout the pedal cycles, see Fig. 5. The active and NMES ERSP charts are relatively similar to each other, both showing oscillations at over 30 Hz, while NMES pedaling shows typical oscillations in around 25 Hz. All the sub-figures demonstrate desynchronization and synchronization patterns in mu or beta frequency bands. However, no significant difference was observed between different pedaling protocols for ERSP or PSD, though NMES pedaling properties tended to be smaller than those of passive pedaling for the mean beta band PSD.

**Table 2:** Clusters of Source Localization for Healthy Subjects

Cluster	Cluster centroid location	Brodmann area	Centroid coordinate	No. of subjects and ICs *
A	L, Medial frontal	BA 6	(-3,-13,50)	10 Subs, 30 ICs
B	L, Cingulate	BA 31	(-8,-55,29)	9 Subs, 29 ICs
C	R, Cingulate	BA 31	(13,-23,33)	8 Subs, 17 ICs
D	R, Cingulate	BA 32	(14,20,36)	7 Subs, 19 ICs
E	R, Precuneus	BA 7	(8,-62,33)	7 Subs, 16 ICs

^*^Total 12 healthy subjects, L: left, R: right, Subs: subjects, ICs: independent components

**Fig. 6:**
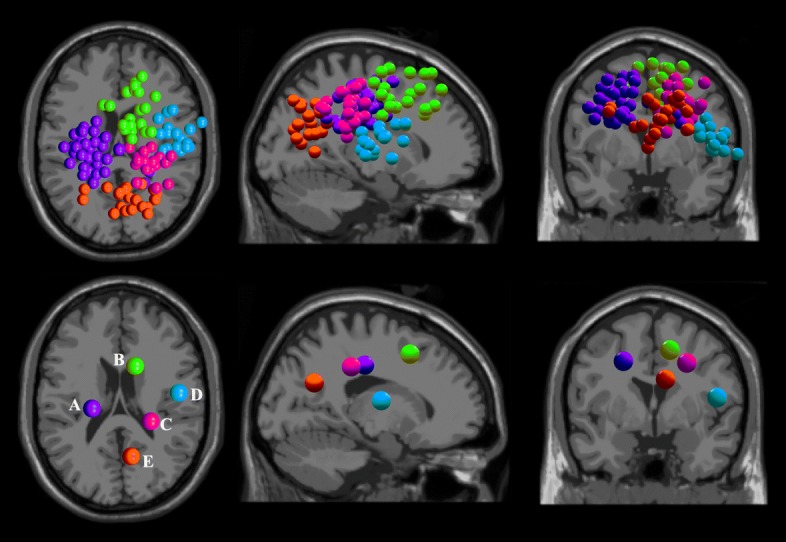
EEG source localization results for sixteen stroke subjects during three pedaling protocols. Five independent component clusters were demonstrated with different colors. The top row gives the dipole locations and the bottom row demonstrated the corresponding cluster centroids, the transverse, sagittal and coronal views (from left to right), A-E, different source clusters centroids in sequence

When it comes to stroke survivors, the detailed cluster centroid location information is demonstrated in Table 3. The source cluster centroids relatively deviated from the mid-area, and the locations are relatively dispersed, see Figure 6 typical cluster D, also cluster A and C. The corresponding motor areas during lower limb pedaling following stroke were shifted according to the EEG source analysis. No significant cortical oscillation difference of ERSP or PSD was found. As the source clusters were not in the same locations, statistical comparison of ICs clusters between healthy and stroke survivors was not conducted.

**Table 3:** Clusters of Source Localization for Stroke Subjects

Cluster	Cluster centroid location	Brodmann area	Centroid coordinate	No. of Subjects and ICs *
A	L, Cingulate	BA 24	(-23,-19,37)	12 Subs, 31 ICs
B	R, Medial frontal	BA 32	(12,15,43)	11 Subs, 21 ICs
C	R, Cingulate	BA 31	(25,-30,36)	11 Subs, 21 ICs
D	R, Precentral	BA 6	(48,-9,10)	9 Subs, 20 ICs
E	R, Precuneus	BA 31	(10, -59, 27)	9 Subs, 20 ICs

^*^Total 16 stroke subjects, L: left, R: Right. Subs: subjects, ICs: independent components

### Directed cortico-muscular coupling during pedaling

Based on the source localization results, this study also investigated the cortico-muscular involvement in pedaling with directed connectivity by generalized partial directed coherence (GPDC). No typical muscle fatigue was reported for across session and subjects by checking the median EMG frequency. Representative peak EEG-EMG and EMG-EEG coherence were observed in beta and low gamma bands, typically around 20 Hz or 30 Hz. As for the group-level comparison of GPDC, muscle dependent GPDC properties were demonstrated for three pedaling protocols. For healthy subjects, GPDC of NMES pedaling is more similar to that of active pedaling for both ascending and descending pathways of selected muscles, suggesting the functional role of NMES in modulating bidirectional GPDC properties. While for stroke survivors, NMES might only promote ascending pathways of GPDC.

**Fig. 7:**
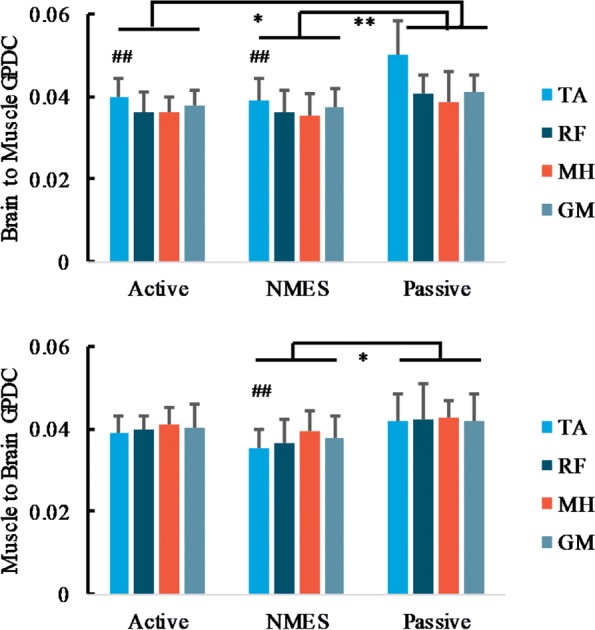
Descending and ascending pathways of GPDC between BA6 cluster and left leg muscles for different pedaling protocols in healthy subjects, average across ICs in the selected pedaling protocol in Table 2, half error-bar= ± 1SEM. Different color represents different muscle. Upper: brain to muscle connectivity, descending pathway, lower: muscle to brain connectivity, ascending pathway. **, *p*<0.01, *, *p*<0.05 for group level (all muscles) comparison between pedaling protocols, *##*, *p*<0.01, *#*, *p*<0.05 for individual muscle comparison with passive pedaling

Two-way repeated measure ANOVA (muscles and protocols) reflects significant main effects of muscles and interaction of muscles*protocols (*F*_(3,81)_=11.934, *p*<0.001, *partial* *η*^2^=0.999, and *F*_(6,81)_=2.371, *p*=0.037, *partial* *η*^2^=0.784 respectively) for GPDC of the descending pathways (brain to muscle). Post-hoc t-test with Bonferroni’s correction found a significant difference between passive and active pedaling, and between passive and NMES pedaling, while no difference was demonstrated between active and NMES pedaling. For individual muscles, GPDC of muscle TA was significantly larger than that of the other three selected muscles (*p*=0.001,<0.001,0.009 respectively). One-way ANOVA found a significant main effect of protocols, and bootstrapping statistics shows a significant difference of GPDC between active and passive pedaling (*p*=0.003), and between NMES and passive pedaling (*p*=0.001). Muscle RF and GM also show a trend of main effect (*p*=0.079,0.118), but did not survive post-hoc tests, see Fig. 7 the upper parts. For ascending pathways (muscle to the brain), no significant main effects were demonstrated, but a significant difference between NMES and passive pedaling was illustrated (*p*=0.011), and ANOVA demonstrates significant or trend of main effect of protocol for TA and RF muscle (*p*=0.116,0.008). For TA muscle, we found a significant difference between NMES and passive pedaling (*p*=0.026), see Fig. 7 the lower parts.

**Fig. 8:**
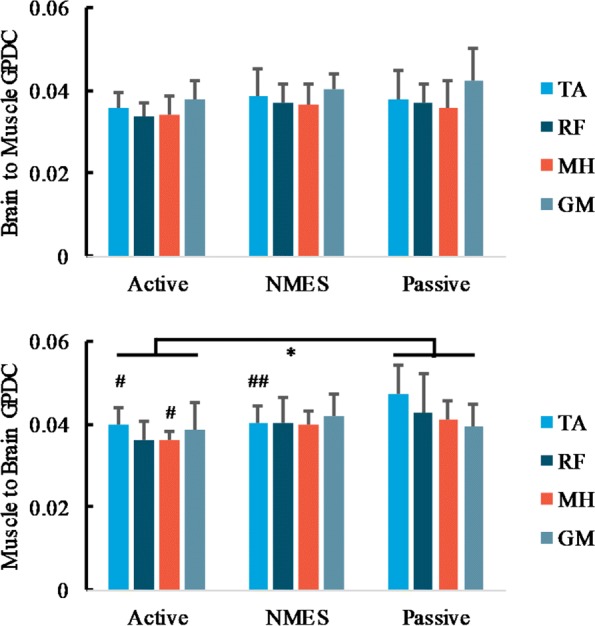
Descending and ascending pathways of GPDC between BA24 cluster and affected leg muscles for different pedaling protocols in chronic stroke subjects, average across ICs in the selected pedaling protocol in Table 3, half error-bar= ± 1 SEM. Different color represents different muscle. Upper: brain to muscle connectivity, descending pathway, lower: muscle to brain connectivity, ascending pathway. *, *p*<0.05 for group level (all muscles) comparison between pedaling protocols, *##*, *p*<0.01, *#*, *p*<0.05 for individual muscle comparison with passive pedaling

Considering chronic stroke survivors, no significant interaction of muscles*protocols of descending pathways of GPDC was shown, post-hoc t-test reports no significant difference between three pedaling protocols (*p*>0.05). One-way ANOVA shows no main effect of protocols for descending pathways for individual muscles, and the post-hot test shows no statistical GPDC difference for different protocols, see Fig. 8 the upper part. Nevertheless, for ascending pathways, we found significant main effects of muscles (*F*_(3,84)_=2.861, *p*=0.042, *partial* *η*^2^=0.666) and trend of muscle*protocol interaction (*F*_(6,84)_=2.083, *p*=0.064, *partial* *η*^2^=0.721). Post-hoc t-test demonstrated a significant difference between active and passive pedaling (*p*=0.034). TA and MH muscles demonstrated significant main effect of protocols (*p*=0.005,*p*=0.015). For TA muscle, statistical difference was demonstrated between passive and active pedaling (*p*=0.02), and between passive and NMES pedaling (*p*=0.009), see Fig. 8 the lower part.

## Discussion

The purpose of the current study was to investigate cortico-muscular contribution during active, passive, and NMES rhythmic pedaling for healthy and chronic stroke survivors. As far as we know, this is the first study to explore directed cortico-muscular coupling in pedaling with general partial directed coherence (GPDC). It was demonstrated that GPDC of NMES pedaling is more similar to that of active pedaling for both healthy and stroke survivors. Moreover, NMES could modulate the GPDC of both ascending and descending pathways for healthy subjects, but it could only induce significant modulatory effects of ascending pathways for chronic stroke survivors.

### Kinematics and EEG source distribution

It is challenging for stroke patients with lower limb impairment to control their lower limb effectively when compared with healthy subjects. Healthy subjects could well maintain the crank torques with no volitional involvement as required. Additionally, pedaling with 20 mA NMES did not significantly increase the crank torques suggesting the stimulation was still sensory-level stimulation instead of motor-level NMES which is typically above 30 mA with 1 ms pulse width [44]. For stroke subjects, the crank torque values were significantly larger than healthy subjects for both NMES and passive pedaling, such results were in line with the previous study in stroke patients [60, 61].

With AMICA source localization and clustering, ICs clusters with more than half subjects involved were determined. The clusters of healthy subjects are relatively close to the middle longitudinal fissure of the cortex and localized to the motor-related area, which was similar to previous studies on gait, robot-assisted movement and pedaling [24, 62, 63]. For both the ICs and cluster centroids, similar movement related cortical activation patterns were shown in this study. The source cluster with the largest numbers for healthy subjects is located in the medial frontal gyrus, which is the corresponding cortical area during pedaling, similar cortico-muscular connectivity patterns were also reported in active and viewed leg movement [64]. Nevertheless, for stroke survivors, the spatial distribution of sources clusters is considerably scattered, this could be accounted for stroke lesion. As also reported by fMRI in stroke patients and healthy controls during ankle dorsiflexion, similar cortical activations in the cingulate and sensorimotor area were illustrated. [65]. Moreover, cortico-muscular coherence study demonstrated that a wider range of cortical regions was involved in affected muscle activities [66].

The most typical cortical source centroid for stroke survivors was located in the cingulate area, which is underneath the BA 6 medial frontal gyrus and is associated with error detection and correction [67]. It is reasonable that the cingulate area has largest response as stroke patients had difficulties in conduct the pedaling experiments. Additionally, the cingulate is related to the balance control process during dynamic movement [62]. These two reasons might explain the different source clusters centroid distribution in stroke when compared to healthy subjects. Moreover, no significant difference between pedaling protocols was reported for the cortical oscillations of ERSP and PSD properties in the selected clusters, which is consistent with the previous study on event-related beta oscillations in active and motor-level NMES induced lower limb movement [17].

### Pathway-specific NMES modulatory effect of cortico-muscular coupling during pedaling

Representative peak source EEG-EMG and EMG-EEG coherence was shown in beta (around 20 Hz) and low gamma band for both healthy and stroke survivors, such patterns were in line with 15-20 Hz peak corticomuscular coherence of ankle dorsiflexion tasks in healthy subjects [68]. Muscle dependent cortico-muscular coupling was demonstrated in this study. The GPDC amplitude of TA muscle varied in different pedaling protocols, suggesting coupling strength between the brain and distally located muscle TA was modulated by NMES, which was also demonstrated in a cortico-muscular coherence study [69]. As for the group-level analysis of muscle*protocol bidirectional cortico-muscular coupling, firstly, no significant difference was presented between active and NMES pedaling for both stroke and healthy subjects. The bidirectional GPDC reflects the functional connection between selected sources and lower limb muscles, and such results were also consistent with previous correlation analysis between active and FES movement [17]. This finding implied that NMES pedaling might be still helpful, even volitional efforts are tough to perform for patients with severe functional disabilities. Secondly, as for the difference between active and passive pedaling, the current study indicated the GPDC of passive pedaling was larger than that of active pedaling which is supported by the fact that active motor contribution to corticokinematic coherence is minimal compared with passive movement [70]. Additionally, the GPDC difference between NMES and passive pedaling protocols was significant for healthy subjects. NMES-induced motor nerve activities was to generate activities similar to the central command [17], while muscle activities in the passive movement were induced by the external pedaling cranks. Electrical pulses on the nerves could elicit action potentials, depolarize the cell membranes of nearby neurons, and further transfer to both muscles and cortical area [71]. Somatosensory input to the motor cortex is important for motor learning and control, and might play critical roles in the motor recovery process [16]. It is reported the NMES-induced motor performance is related to strong sensory input and facilitated motor control process [19], and sensory-level NMES fosters motor imagery performance for healthy subjects [72]. Previous fMRI study during three state ankle dorsiflexion (active, passive, and electrical stimulation) proved that significantly larger numbers of voxels were activated during active and electrical stimulation conditions [73]. These results also supported our findings, future studies might employ diffusion MRI techniques to validate the pathway difference in dynamic movement environment.

As stroke survivors cannot perform stable stance or gait activities, stationary pedaling was conducted in the current study, which shares a similar group of muscle activation as waking [9]. Future studies might investigate the cortico-muscular activation of walking or other complex movements with advanced robot-assisted techniques for patients with movement disorders. Additionally, though great efforts were applied in various lower limb human locomotion tasks like gait, treadmill walking, and balance maintenance [26, 74, 74, 75], the body position-specific and task-specific cortico-muscular response was rarely discussed. It should be accounted for the deep somatotopy of lower limbs [76], subsequent analysis should consider the task-dependent and position-dependent motor control mechanisms during fine lower limb locomotion activities.

Furthermore, there is no significant difference between any of the three protocols for descending pathways of cortico-muscular coupling for chronic stroke survivors. It indicates that the brain to muscle GPDC is relatively stable in the three pedaling protocols for the selected muscles and the source clusters. While both the ascending pathways and descending pathways are modulated by the 20 mA NMES for healthy subjects. Stroke lesions decrease the corticospinal tract integrity for especially the descending efferent pathway [77], which induced abnormal motor synergy and disordered motor control [78]. It is plausible that the motor thresholds of stroke lower limb muscles are larger than that of healthy subjects due to stroke-induced spasticity, muscle changes, and supraspinal abnormality [28]. Longer periods of electrical stimulation might induce more sustained changes for especially stroke subjects [40]. Moreover, NMES could still activate ascending volley of afferents which could promote interneurons and motoneurons synaptic plasticity and induce sensory axons activation after stroke [15]. Additionally, the recruited central orthodromic motor signals following stimulation might be limited as a result of stroke lesion [79]. These could partially explain the current findings that NMES only modulates ascending pathways in chronic stroke subjects with moderate motor functions. Future study with larger NMES amplitude might be necessary to validate the hypothesis and further determine the NMES threshold to induce functional changes in bidirectional pathways of motor control. NMES with larger amplitude might be required for clinical rehabilitation applications following this line. The current findings might assist in distinguishing the underlying mechanisms of NMES in facilitating motor control and learning in stroke rehabilitation. Future studies might consider stimulating both the motor cortex and peripheral muscles to acquire parallel recovery of motor and sensory pathways in chronic stroke patients.

There are several limitations for the current study. No gender and age-matched healthy subjects were included in this study, although we did not perform a statistical comparison of directed cortico-muscular coupling between stroke and healthy subjects. The exact lesion locations from MRI/DTI were not available for some subjects. Further studies should consider the influence of stroke lesion on the NMES modulatory effects, diffusion MRI and other neuroimaging techniques might be necessary to explore the cortico-spinal tract and investigate potential modulatory effects on the pyramidal tracts and motor cortex [80]. The complex crosstalk in the four EMG measurement channels made it challenging to remove the 40 Hz NMES artifact in EMG signals [81], the frequency band above 35 Hz was not considered. Future study should remove all the stimulation artifacts and perform a systematic analysis of the EMG signals during various pedaling. Moreover, due to small sample size, only the source clusters with the largest amount were included for statistical analysis, and it is hard to compare the source clusters between healthy and stroke survivors directly owing to the inconsistent source dipoles and cluster centroids.

## Conclusion

In summary, we investigated the directed cortico-muscular coupling by generalized partial directed coherence (GPDC) in rhythmic pedaling with neuromuscular electrical stimulation (NMES) at the sensory level for both healthy and stroke survivors. Bidirectional GPDC of NMES pedaling was more similar to that of active pedaling instead of passive pedaling, and this might suggest the functional role of NMES in facilitating motor control of affected limbs to that of the normal state. Moreover, the GPDC in different pedaling protocols could be muscle dependent. Especially, for selected muscles like Tibialis anterior, the NMES might modulate GPDC of both descending and ascending pathways for healthy subjects. Nevertheless, sensory-level NMES might only modulate the ascending sensory pathways for stroke survivors, and this might facilitate understanding the potential modulatory mechanisms of NMES in promoting motor control and learning in stroke rehabilitation. This study has far-reaching implications for future applications of NMES based stroke rehabilitation protocols.

## Data Availability

The raw data, including the EEG/EMG was disclosed for individual subjects. It has been stated in the consent approved by the Joint Chinese University of Hong Kong-New Territories East Cluster (CUHK-NTEC) Clinical Research Ethics Committee that the results might be published, but the individual data would be kept confidentially for subjects.

## Abbreviations

AMICA: Adaptive mixture independent component analysis
CNS: Central nervous system
EEG: Electroencephalogram
EMG: Electromyography
ERSP: Event-related spectral perturbation
FMA-LM: Fugl-Meyer assessment lower limb
GPDC: Generalized partial directed coherence
ICs: Independent components
MEPs: Motor evoked potentials
MH: Medial hamstring
NMES: Neuromuscular electrical stimulation
PSD: Power spectral density
RPM: Revolutions per minute
TA: Tibialis anterior
TDC: Top dead center
